# The Flatland Fallacy: Moving Beyond Low–Dimensional Thinking

**DOI:** 10.1111/tops.12404

**Published:** 2018-12-21

**Authors:** Eshin Jolly, Luke J. Chang

**Affiliations:** ^1^ Computational Social Affective Neuroscience Laboratory Department of Psychological and Brain Sciences Dartmouth College

**Keywords:** Dual‐processing, Computational, Social, Decision‐making, Psychological education

## Abstract

Psychology is a complicated science. It has no general axioms or mathematical proofs, is rarely directly observable, and is the only discipline in which the subject matter (i.e., human psychological phenomena) is also the tool of investigation. Like the Flatlanders in Edwin Abbot's famous short story (1884), we may be led to believe that the parsimony offered by our *low‐dimensional* theories reflects the reality of a much *higher‐dimensional* problem. Here we contend that this “Flatland fallacy” leads us to seek out simplified explanations of complex phenomena, limiting our capacity as scientists to build and communicate useful models of human psychology. We suggest that this fallacy can be overcome through (a) the use of quantitative models, which force researchers to formalize their theories to overcome this fallacy, and (b) improved quantitative training, which can build new norms for conducting psychological research.

## Abstract 2

In rebellion against low‐dimensional (e.g., two‐factor) theories in psychology, the authors make the case for high‐dimensional theories. This change in perspective requires a shift towards a focus on computation and quantitative reasoning.

1

*Yet I exist in the hope that these memoirs, in some manner, I know not how, may find their way to the minds of humanity in Some Dimension, and may stir up a race of rebels who shall refuse to be confined to limited Dimensionality*.

—Edwin A. Abbott, *Flatland: A Romance of Many Dimensions* (1884)

## 2 Introduction

**Figure 1:**
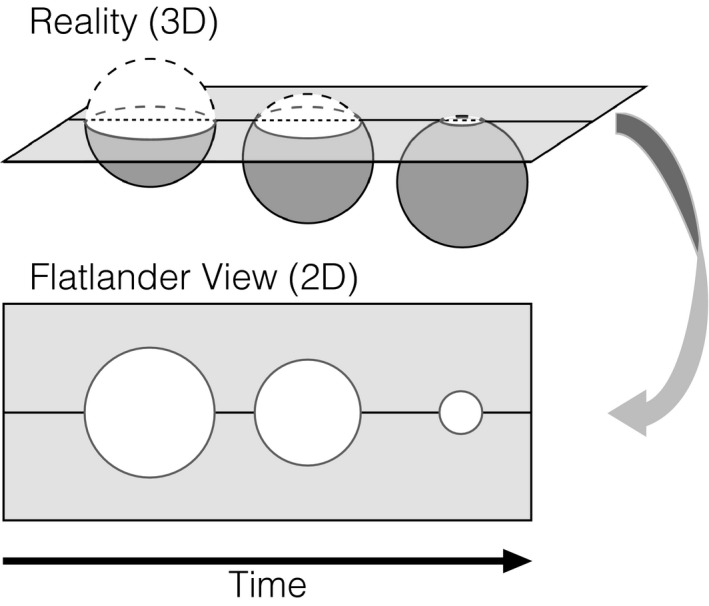
In Abbott's novella, A. Square cannot perceive his world as anything other than two dimensional. From his limited perspective (“Flatlander View”; bottom), a three‐dimensional entity (sphere) appears to be changing sizes before him (growing and shrinking circle). In reality (top), this entity is simply moving through a lower‐dimensional plane, but A. Square's limited perspective leads to a false conclusion about the nature of reality. For similar reasons, psychological scientists may falsely conclude that the number of dimensions that accurately characterize psychological phenomena is sufficiently small, viewing the world like Flatlanders, even if in reality the complexity of psychological phenomena is high dimensional.

Few works consider the nature of perception and dimensionality as elegantly as Edwin Abbott's (1884) novella *Flatland: A Romance of Many Dimensions*. The narrator of the story, A. Square, lives in a world full of “Flatlanders,” who are incapable of perceiving or even conceiving of a reality that exists beyond two dimensions. However, after a visit from a “Stranger” (a sphere) A. Square comes to appreciate how complex and high dimensional the world really is. Ultimately, he is imprisoned for his heretical beliefs after trying to teach his colleagues about his revelations. Abbott's key insight was that creatures with limited perceptual capacities (i.e., seeing in only two dimensions) come to *reason* in a limited way, ignoring the complexity of the world and truly believing their perceptions to be veridical (Fig. 1). Much like Flatlanders, humans exhibit strong biases in their reasoning about a complex and high‐dimensional world due to finite limitations on their cognitive capacities. For this reason, we posit that psychological researchers, constrained by these limitations, may come to view the complexities of psychological life in similarly limited ways.

Human psychology is rife with complexity, the product of an immensely high‐dimensional space characterized by interactions between trillions of neural connections, billions of unique individuals, and dynamic changing contexts spanning thousands of years of history. Despite this complexity, the majority of theoretical developments in psychological research have consistently converged on producing a number of low (typically two) dimensional/factor theories/process models of human mental life. Since at least the early work of Plato (Evans & Frankish, 2009), this manner of characterizing the mind has come to dominate our understanding of *emotion* (Damasio, 1994; Davidson, 1993; Ochsner, Bunge, Gross, & Gabrieli, 2002; Posner, Russell, & Peterson, 2005; Russell, 1980; Schachter & Singer, 1962; Zajonc, 1980), *social cognition* (Chaiken & Trope, 1999; Cuddy, Fiske, & Glick, 2008; Gray, Gray, & Wegner, 2007; Gray & Wegner, 2009; Greenwald & Banaji, 1995; Haslam, 2006; Mitchell, 2005; Saxe, 2005; Todorov, Said, Engell, & Oosterhof, 2008; Waytz & Mitchell, 2011), *moral judgment* (Greene, Sommerville, Nystrom, Darley, & Cohen, 2001; Rand, Greene, & Nowak, 2012), *learning* (Daw, Niv, & Dayan, 2005; Frank, Cohen, & Sanfey, 2009; Poldrack et al., 2001; Poldrack & Packard, 2003), c*ognitive control* (Heatherton & Wagner, 2011; Hofmann, Friese, & Strack, 2009; McClure, Laibson, Loewenstein, & Cohen, 2004; Schneider & Shiffrin, 1977; Shiffrin & Schneider, 1984), *decision‐making* (Chang & Sanfey, 2008; Dijksterhuis, Bos, Nordgren, & van Baaren, 2006; Kahneman, 2003; Sanfey & Chang, 2008; Sloman, 1996; Wilson & Schooler, 1991), and *reasoning* (Epstein, 1994; Evans, 2003; Stanovich & West, 2000). How could something as complex as the human mind be consistently described in two dimensions, irrespective of the mental faculty under consideration? Although these theories have provided a bedrock for empirical investigation, we argue that rather than reflecting a rich characterization of the complexity of human psychology, they instead reflect a simplistic view of our scientific understanding (Flatland fallacy)—a product of the limits of our cognition.

In this paper, we outline several reasons why we believe psychologists consistently converge on two‐factor solutions to characterize our understanding of human psychology. We argue that these conclusions arise from our limited cognitive capacities, social norms ubiquitous in the field of psychology, and our reliance on low‐bandwidth channels to communicate research findings (e.g., natural language and simple visualizations). We suggest that moving beyond low‐dimensional thinking requires formalizing psychological theories as *quantitative* computational models capable of making precise predictions about cognition and/or behavior, and we advocate for improving training in technical skills and quantitative reasoning in psychology.

## 3 Why does the Flatland fallacy happen?

Understanding why the Flatland fallacy occurs requires examining both biases and limitations in human cognition as well as cultural norms in research and training in psychology. Specifically, we propose four main reasons why this fallacy occurs: (a) biases in the feeling of understanding; (b) limitations of human cognition; (c) over‐reliance on traditional experimental design and analytic approaches; and (d) limitations in our ability to communicate complex concepts. Because psychology researchers have the unique privilege of being members of both the matter of study and those conducting the study, it is critical that the products of our science not be constrained by the limits of our own psychology (Meehl, 1954).

### 3.1 Feelings of understanding

Much like Abbott's A. Square feels that a two‐dimensional existence is a complete account of his universe, humans are prone to a “folk understanding bias”—the sensation that simplistic explanations lead us to believe we truly understand more complex phenomena. Prior work in cognitive science and philosophy has illustrated how individuals can fall victim to cognitive biases that lead them to believe their actual understanding of phenomena exceeds their true understanding of phenomena. For example, individuals often report a high feeling‐of‐knowing despite their inability to accurately recall previously learned information (Koriat, 1993). When individuals are tested on their ability to explain how a system works (e.g., a quartz watch), they tend to report an overestimate of their knowledge until they are asked to provide a specific explication (Rozenblit & Keil, 2002). In other words, individuals create mental placeholders of elaborate, in‐depth explanations (e.g., essences and hidden mechanisms) that give rise to a feeling of certainty and understanding, even when limited understanding exists (Medin, 1989; Strevens, 2000). Because these approximations can provide basic explanations as to how a system works, they are initially insightful, leaving people with the sensation that they know more than they really do (Rozenblit & Keil, 2002).

A critical observation is that this bias is exacerbated when individuals are asked to explain systems that are highly opaque, that is, have poor observability of their inner workings (Rozenblit & Keil, 2002). Unfortunately, the complexity of psychological science lies almost entirely in its lack of transparency; mental processes are not directly observable, only inferrable through observations of behavior and their correlations with biological functioning.

This bias likely originates from our strong motivation to understand and find meaning in our experiences and the world as a whole (Cohen, Stotland, & Wolfe, 1955). For this reason, it makes sense to favor simplistic and easily understandable theoretical conclusions over complex and complete accounts of phenomena, even if they are only weakly supported by experimental data. Simple explanations provide some uncertainty resolution even if they paint an incomplete picture (Pinker, 1999; Webster & Kruglanski, 1994). Consequently, researchers may be collectively at risk for pursuing a psychological science that they can “understand,” irrespective of whether that science offers robust predictive accuracy.

### 3.2 Limitations of cognitive capacity

Limitations in the cognitive capacities and motivations of individuals offer another explanation for the Flatland fallacy. It is well established, for example, that humans are not supercomputers who always calculate mathematically optimal solutions for the problems they face (Gigerenzer & Goldstein, 1996). Rather, our brains are the product of specific evolutionary constraints such as physical *size*—they need to be small enough to permit live births and allow us to locomote; *speed*—they need to support processing that can occur on finite time scales; and *energy*—their energy demands cannot exceed our metabolic abilities (Montague, 2007). For this reason, the notion of bounded rationality has been instrumental in characterizing how the mind processes information quickly and reasonably accurately (Simon, 1957). There are many well‐known examples, such as our limited capacity to simultaneously manipulate large chunks of information (Miller, 1956), process multiple attentional tasks (Pashler, 1994), and uniquely represent person‐identity information without relying on feature similarity such as stereotypes (Mervis & Rosch, 1981; Smith & Zarate, 1992).

We believe that the key psychological limitation underlying the Flatland fallacy is our inability to reason in more than a few dimensions, particularly in contexts that require integrating multiple sources of information together. Individuals tend to default to simpler, general, heuristic‐like strategies that serve to make such reasoning more cognitively tractable. These strategies often constitute lower‐dimensional approximations (e.g., two or three) of far more complex information landscapes, which raises the possibility that even the process of conducting scientific research can be similarly marred by the limits of our cognition. We outline three examples of how lower dimensional approximations impact how we make judgments and decisions.

#### 3.2.1 Judgment

Many real‐world settings involve situations in which individuals are faced with the task of making judgments by combining a large number of potentially relevant factors (e.g., clinical evaluations). A large body of work has consistently demonstrated that humans make judgments using just a handful of dimensions (Brunswick, 1952) rather than considering all the available information on hand. In particular, individuals over weight or under weight the relative importance of specific factors or simply ignore seemingly irrelevant information in favor of simplified evaluation criteria, such as when estimating school admissions, personality metrics, and even criminal evaluations (Dudycha & Naylor, 1966; Karelaia & Hogarth, 2008; Meehl, 1954). In other words, individuals rely on heuristics to simplify the space of information under consideration, especially when this space is very large or shares a nonlinear relationship with an outcome (Deane, Hammond, & Summers, 1972; Karelaia & Hogarth, 2008). Given the robustness of this evidence, there has been a strong call to incorporate statistical models that can integrate more dimensions in place of solely relying on clinical judgment to overcome these cognitive limitations (Dawes, 1971; Dawes, Faust, & Meehl, 1989; Meehl, 1954).

#### 3.2.2 Decision‐making

Dual‐process theories have a rich history of characterizing human decision‐making (Sanfey & Chang, 2008). These accounts have been used to explain how we can switch between fast, intuitive, and reflexive modes of thinking to slow deliberative calculations (Kahneman, 2003, 2011) and also how emotions and cognitive deliberation might be integrated when making decisions (Chang & Sanfey, 2008; Chang, Smith, Dufwenberg, & Sanfey, 2011; Greene, Nystrom, Engell, Darley, & Cohen, 2004). In addition, there appears to be an upper limit on the number of attributes that can be simultaneously considered when making a decision between different choices (Ashby, Alfonso‐Reese, Turken, & Waldron, 1998; Payne, 1976). Faced with a large number of factors to consider, individuals appear to act in an adaptive manner, falling back on heuristic shortcuts rather than considering all of the available information on hand (Payne, Bettman, & Johnson, 1993; Simon, 1987). In other words, humans tend to make high‐dimensional problems more cognitively tractable by considering lower dimensional perspectives—specifically, through the use of heuristic strategies that entail ignoring potentially relevant factors (Gigerenzer & Brighton, 2009).

#### 3.2.3 Conditional reasoning

Even in more socially interactive contexts that require individuals to consider the motivations of *others*, individuals exhibit consistent limits on their cognitive abilities. A large body of work in game theory and behavioral economics has demonstrated that individuals are limited in their depth of strategic reasoning (Camerer, Ho, & Chong, 2015). In this work, individuals compete or coordinate with each other within an economic game. Players' strategies in these games allow for optimizing their own payoffs while also considering the strategy utilized by other players. Interestingly, individuals are rarely able to reason more than two steps ahead of other individuals, (i.e., more than two levels of such conditional reasoning: “I think that you think that I think”) (Camerer, Ho, & Chong, 2004; Griessinger & Coricelli, 2015; Stahl & Wilson, 1995). Even in non‐strategic contexts, individuals have been shown to exhibit limits on the amount of recursive reasoning they are capable of, such as during theory‐of‐mind tasks that require inferring the motives of fictionalized agents (e.g., Happé, 1994) or, more generally, parsing language comprised of numerous embedded clauses (Karlsson, 2010). Across a large number of spoken languages, for example, this type of syntactic recursion (e.g., “a car the man the woman the boy saw drove fast”) rarely exceeds two levels of depth and even in written text rarely exceeds three levels of depth (Karlsson, 2007). Because understanding conditional complexity quickly becomes incredibly difficult, individuals fall back on using simple heuristic strategies and generalized decisions rules in lieu of making more optimally rational choices (Camerer, Johnson, Rymon, & Sen, 1993; Costa‐Gomes, Crawford, & Broseta, 2001).

Taken together, these findings suggest that in the face of complex information processing, individuals intuitively converge on strategies that reduce the number of factors (dimensions) under consideration to make cognitive problems more tractable. Because the dimensionality of factors necessary for understanding human psychology is incredibly high, psychological researchers may be focusing on far fewer dimensions than what actually comprise the mind. That is, scientists may intuitively converge on establishing low‐dimensional theories (e.g., dual process models) because they allow for a reduction of the diverse set of factors relevant to building a comprehensive account of psychological processing.

### 3.3 Cultural norms

Together with individual biases and cognitive limitations, we believe that methodological traditions within the field of psychology have built a pedagogy that supports the Flatland fallacy. Heavily inspired by Ronald Fisher's iconic Statistic Methods for Research Workers (Fisher, 1925) and deeply embedded in undergraduate training in psychology, there is a strong persistent cultural tradition of academic psychology's reliance on a specific type of experimental design and statistical analysis to test hypotheses: two‐dimensional factorial designs (i.e., two‐way analysis of variance; anova) evaluated via null hypothesis significance testing (NHST).

Although psychology does not have a generally agreed–upon core curriculum, at minimum almost all psychology undergraduate programs require that students take an introductory psychology course and one or more courses in statistics or research methods (Stoloff et al., 2009). Introductory statistics courses typically introduce basic concepts of inferential statistics, culminating in an introduction to two‐way anova. Research methods courses primarily emphasize making causal inferences using 2 × 2 factorial designs. In addition, approximately 40% of psychology programs require taking five courses in specific topics of psychology (e.g., abnormal, developmental, cognitive, social, biological) and a capstone course that results in a culminating experience for the psychology major (Stoloff et al., 2009). This limited training in statistical theory and psychological measurement persists into graduate training. A survey conducted in 1990 of psychology doctoral programs found that statistical training in most programs was highly similar to what it had been 20 years prior with 73% of programs providing in‐depth understanding of “old standards of statistics” predominantly ANOVA, but only 21% provided more advanced training such as multivariate procedures (Aiken et al., 1990). A follow‐up study 20 years later reached nearly identical conclusions with 80% of training still devoted to anova and a continual general decline in measurement training (Aiken, West, & Millsap, 2008). In particular, Aiken et al., (2008) note the decline in coverage of measurement techniques (median 4.5 weeks of total PhD curriculum) with many programs offering absolutely no training in test theory or construction.

We believe this pedagogy not only trains generations of psychological scientists to pursue empirical investigations that favor simple factorial designs, but also teaches them to *think* about psychological science in a low‐dimensional way. Consequently, the standard approach for inferential reasoning in psychology produces theoretical ideas that are limited to measuring mean differences between variables manipulated along a handful of psychological dimensions, thereby perpetuating the Flatland fallacy.

Although this approach provides an accessible starting point for the evaluation of psychological phenomena, significance testing of experimental manipulations alone does not constitute a formal model of human psychology and cannot be used as evidence for such (Bolles, 1962). The pervasive danger of relying on this approach when testing psychological theory is that it reinforces the generation of weaker, less specific, and more nebulous theories, particularly in a growing era of “big data” (Meehl, 1990; Van Horn & Toga, 2014). This methodological paradox was elegantly articulated more than 50 years ago by Paul Meehl (Meehl, 1967). Whereas increasing experimental power constitutes a *more difficult* test that a quantitative theory must pass to remain viable (e.g., a mathematical model in physics), the exact opposite is true of psychological research, which is primarily concerned with utilizing NHST to detect arbitrary non‐zero differences between experimental conditions. Because measurement error decreases with increasing power and precision, smaller non‐zero differences will be *necessarily* more detectable in the limit of NHST. This leads psychological researchers to conclude that a statistically significant result of a trivially small difference provides support for a given theory. By instead developing theories as the “prediction of a *form* of function (with parameters to be fitted)” or “prediction of a quantitative magnitude (point‐value),” the band of tolerance around theoretical validity *decreases* as experimental fidelity increases (Meehl, 1967). In other words, using NHST to evaluate theories formalized as models that make specific quantitative predictions ensures that theories must be *specific* in order to remain robust.

**Figure 2:**
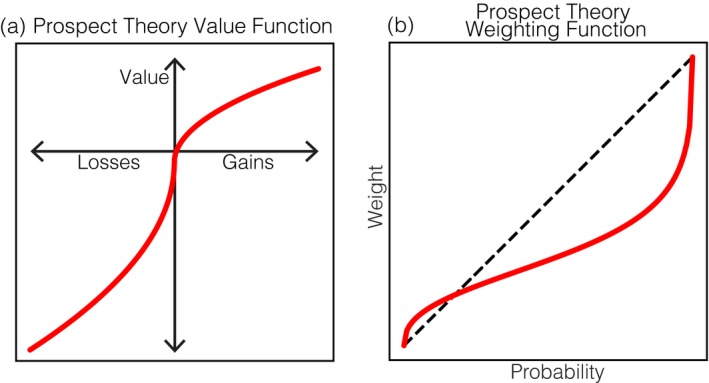
(a) Prospect theory describes a mathematical *function* that maps between financial gains and losses and the subjective value that individuals experience. This function can be used to predict how an individual will make decisions and explains their tendency to exhibit risk‐seeking or risk‐averse behavior. (b) A central part of this theory is a functional account of how individuals treat probability values when making decisions, illustrating how small probability events receive more consideration than large probability events when making choices.

A classic example of the benefit of such an approach in psychology can be found in prospect theory (Kahneman & Tversky, 1979). Prospect theory outlines the form of a function that describes how the quantitative gain or loss that an individual incurs is mathematically transformed into the subjective *value* he or she feels (Fig. 2a). Critically, this theory defines a weighting function (Fig. 2b) for outcomes that cannot only be used to predict individuals' decisions, but also captures the asymmetry that occurs between changes in income framed as gains or losses. This model differs drastically from conventional approaches discussed previously in that it can be used to make point predictions about individuals' behavior and attitudes in a wide variety of decision contexts (Tversky & Kahneman, 1974).

### 3.4 Communicating complexity

Lastly, we consider how a pervasive communication problem endemic to the field of psychology as a whole gives rise to the Flatland fallacy. Central to this issue is that collectively, psychological researchers lack a *lingua franca* that enables us to communicate about the complexities of our field. While numerous succinct psychological constructs exist to describe complicated ideas (e.g., “stereotype threat” (Steele & Aronson, 1995), “affective forecasting” (Gilbert & Wilson, 2000), this terminology is fundamentally ad hoc, making it challenging to develop a shared and extensible framework for the communication and development of new ideas. In contrast to social science fields that rely on verbal descriptions of phenomena (e.g., psychology) (Watts, 2017), scientific disciplines that have methods of communicating about high‐dimensional problems using mathematics (e.g., physics, computer science, economics), concrete physical models (e.g., biology, astronomy), or their own notation (e.g., chemistry) may be less susceptible to the Flatland fallacy. We argue that in the absence of a foundational dialect, psychology has suffered from an inability to communicate complexity, instead generating theories which make crude and imprecise predictions that may be difficult to falsify (Meehl, 1990). As a result, psychological discourse is more like a pidgin language than a *lingua franca*: simplified mixtures and non‐specific generalizations that make it challenging to build a cumulative science.

This communication problem is particularly exacerbated by the limited number of dimensions available to psychologists to visualize their findings. In theory, data visualizations provide a useful tool for detecting patterns and interpreting research findings. Several recent technical and software advances in this area can provide aid in overcoming this limitation by learning “low‐dimensional embeddings” of high‐dimensional data that attempt to preserve distance (Zhang, Huang, & Wang, 2010), such as t‐SNE (t‐distributed stochastic neighbor embedding) (van der Maaten & Hinton, 2008) and UMAP (uniform manifold approximation and projection) (McInnes & Healy, 2018). Both of these algorithms are implemented in easy‐to‐use open source software packages such as hypertools (Heusser, Ziman, Owen, & Manning, 2018) However, even with such advanced techniques, graphical representations of research findings are typically limited to about three dimensions.^1^ This is particularly problematic in the absence of a formal framework to build a cumulative science (e.g., mathematics), as psychologists are only able to interpret experiments that independently manipulate a few dimensions (e.g., a three‐way ANOVA). This creates a tension between theoretical interpretability and theoretical extensibility. Because psychological science lacks a formal discourse, researchers are motivated to design experiments around low‐dimensional theory testing and simple visualizations, but because experiments designed to test low‐dimensional theories are not by themselves extensible, psychological science exists as a patchwork of disparate, loosely connected ideas. Even recent advances in “theory mapping” (Gray, 2017), which provide organizational instructions as to how to connect psychological theories to each other, are underspecified and underconstrained (Newell, 1973) because they fail to provide comprehensive parameterized *models* of psychology that can be used to make useful quantitative predictions (Yarkoni & Westfall, 2017) about behavior or cognition.

## 4 What are some solutions?

We believe that overcoming the Flatland fallacy requires advances in the methodological approach that psychological scientists take toward their own work and also pedagogical changes that train future generations of researchers to build upon and extend extant work. First, we highlight the role that *computational models* can play in enabling researchers to overcome the biases and limitations in their own cognition, as well as enabling multiple researchers to work together to build a more cumulative science. Second, we outline some suggestions for the improvement of *psychological training*, stressing the importance of teaching students the fundamentals of mathematics and computer programming. We believe that both of these approaches are required to mature the field of psychological science.

### 4.1 Computational models

Formalizing psychological theories using computational models provides a way to overcome the Flatland fallacy through the consideration of *high dimensional* explanations of psychological phenomena. Indeed, recent methodological advances in neuroscience have demonstrated how information in the brain is encoded with incredibly high dimensionality with respect to both space and time (Haxby, Connolly, & Guntupalli, 2014). We believe the use of computational models will likewise better enable researchers to capture this complexity within psychological theories. Most psychological researchers are already familiar with regression as an instance of a *statistical model*, specifically a linear one that combines features according to a set of weights, in order to predict the value of a dependent variable. We encourage researchers to think of models in more general terms: a general mathematical function that transforms inputs into specific outputs.^2^ In this way, models can be likened to cooking recipes that describe how to best combine ingredients into prepared foods (Crockett, 2016). We believe this analogy can help elucidate how models can play a critical role in overcoming the Flatland fallacy.

Imagine eating a piece of cake: observing the colors and designs that draw your eye, tasting the different flavors and textures as you take a bite, and experiencing the way numerous ingredients come together to create a delightful sensory experience. What ingredients determine the colors you see and the flavors you taste? Was that a hint of vanilla? Does the frosting contain cardamom? You might attempt to recreate this sensory experience in your own kitchen, combining numerous ingredients in different proportions, transforming those ingredients through baking at different temperatures, until you can reliably reproduce a tasty baked good. Through a painstaking trial‐and‐error process you might converge on a *recipe* for *how* to combine and transform a set of raw ingredients into a finished product. In much the same way that recipes serve as a set of instructions for combining specific ingredients together that produce a cake, models provide mathematical formulations for combining different input *features* together that produce an outcome. These features can span a space that comprises only a few dimensions just as it takes only takes a handful of ingredients to make a pound cake. They can also, however, be incredibly enumerate and involve complex nonlinear interactions, just as a dobos torte requires intricately interleaving many layers of a thin sponge cake with buttercream. Indeed, with recent advances in deep learning, some models can comprise millions of distinct features organized into hidden layers that learn many different ways to filter and transform input data (e.g., Huang, Sun, Liu, Sedra, & Weinberger, 2016). Yet despite this wide range of complexity, researchers need not manipulate so many inputs at once singlehandedly. Instead, they can rely on a model which serves as powerful and reliable assistive tool.

At their core, recipes are comprised of ingredients, proportions of ingredients, and instructions for how to combine ingredients to produce a finished product. Like recipes, models are comprised of *features* that are scaled by *weights* and combined in a specific *formula* to produce a *prediction* (Table 1). Central to our argument is that models serve as tools to both reason and communicate about *high–dimensional* spaces. Models allow researchers to consider what dimensions of a problem are most relevant and predict outcomes based on complex sets of interactions. Models also allow researchers to build intuitions about their components through simulation and application to novel datasets (Yarkoni & Westfall, 2017), akin to children taking devices apart to figure out how they work. Moreover, models can be shared between researchers, permitting the collective development of a cumulative science whereby weak or redundant theories are pruned and robust, predictive theories are retained.

**Table 1:** Relationship between models and recipes

Recipe	Model	Purpose
Ingredients	Inputs/features	Characterize the possible building blocks (dimension) necessary to create an output
Proportions	Weights/parameters	Characterize the relative importance of each input
Instructions	Formula/model specification	A describe a set of mathematical rules for ways in which inputs should be combined
Product	Prediction	The output consequent of combining inputs according to a set of rules with a set of fixed levels of importance

A key property of formalizing psychological theories as computational models is that it enables researchers to share their ideas in *extensible* ways. In contrast, the significance result of an anova is fundamentally useless to other researchers trying to extend prior theoretical work (Schmidt, 1996): *p*‐values and effect sizes speak to the likelihood of the data under a null‐hypothesis but are unable to provide precise predictions (point‐estimates) about new data in new contexts (Meehl, 1967) or evaluate the predictive capabilities of competing ideas (i.e., model selection/comparison). At best, researchers will only be able to devise novel experiments to test contextual moderators on the coarse treatment effects that a theory predicts (Van Bavel, Mende‐Siedlecki, Brady, & Reinero, 2016). On the other hand, models are simply recipes for combining inputs to generate predictions, and they can therefore be easily applied to novel contexts using new data. For example, we have developed models of how emotions (Chang & Smith, 2015; Chang et al., 2011), social norms (Chang & Sanfey, 2013; Sanfey, Stallen, & Chang, 2014), and inferences about others (Chang, Doll, van't Wout, Frank, & Sanfey, 2010; Fareri, Chang, & Delgado, 2015; Sul, Güroğlu, Crone, & Chang, 2017) can predict decisions to cooperate. In addition, we and others have developed models for how activity in different brain regions might be combined to produce an affective response (Chang, Gianaros, Manuck, Krishnan, & Wager, 2015; Eisenbarth, Chang, & Wager, 2016; Krishnan et al., 2016; Wager et al., 2013). Perhaps one of the best examples of how models can lead to a cumulative and extensible study of cognition can be found in neurally inspired connectionist models. This work strives to synthesize neuroscience findings into high‐dimensional models of how the brain implements specific cognitive functions such as learning (O'Reilly & Frank, 2006; O'Reilly, Frank, Hazy, & Watz, 2007), memory (Norman & O'Reilly, 2003), and decision‐making (Frank & Claus, 2006). These models have been integrated into a programming framework (e.g., Leabra) that provides a holistic architecture capable of making precise predictions of a wide array of cognitive processes (O'Reilly, Hazy, & Herd, 2016). The advantage of these types of quantitative models is that they permit precise evaluation of how *sensitive* a model is for capturing a psychological construct, as well as how *specific* a model is to a given construct as compared to other psychological states and processes. Further, these models are shareable and extensible by other researchers, allowing them to directly build upon previous work to test how a given model, such as a marker of negative affect, responds in a new experimental condition and generalizes to a novel context (e.g., Gilead et al., 2016; Krishnan et al., 2016). This process of iterative construct validation through model sharing and testing on many types of data is critical for developing a cumulative science of what comprises psychological states and how they are encoded and represented in the brain (Woo, Chang, Lindquist, & Wager, 2017).

### 4.2 Improved quantitative training

Although computational modeling offers an approach for uncovering psychological phenomena in higher dimensions, it requires a dramatic reform in the way the discipline of psychology carries out quantitative training. Instead of providing a limited introduction to inference using statistics and research design and separately prioritizing the memorization of psychological effects in different domains of psychology, we believe there should be increased emphasis on teaching technical skills and a better education of data‐driven inferential reasoning within all psychology courses. Moreover, psychology's curriculum could be expanded to adapt to the recent technological advances that have resulted in the exponential growth in the collection of data via the Internet, online commerce, mobile sensing, and so on (Griffiths, 2015; Lazer et al., 2009; Yarkoni, 2012). Beyond the narrow domain of academic psychology, there already exists intense demand for skilled workers who can gain insights about human behavior from data in almost every industry, including government, journalism, business, and healthcare. We believe that with improved training, psychologists could become increasingly involved in these efforts, ultimately providing an opportunity to inform an array of diverse and important industries and issues.

To do so will require reimagining training in psychology. Working with large, complicated datasets requires basic training in areas traditionally associated with computer science and informatics, including programming, algorithms, databases, and computing. This requires extending basic education of statistical training to include skills such as data manipulation, generating predictive models, machine‐learning, natural language processing, graph theory, and visualization (Montag, Duke, & Markowetz, 2016; Yarkoni, 2012; Yarkoni & Westfall, 2017). These types of competencies comprise an emerging growth of applied statistics or “data science” programs (Anderson, Bowring, McCauley, Pothering, & Starr, 2014). We believe that increased emphasis on training basic technical and quantitative skills will improve the ability of psychology majors to participate in the enormous endeavor of understanding human behavior from data. However, we are certainly not advocating that psychology majors should additionally pursue an accompanying degree in statistics or computer science. Instead, we recommend that training programs in psychology consider adding additional requirements to curricula (e.g., programming and computing for psychologists & advanced statistics), providing better integration of data‐driven inferential reasoning skills into existing curricula (e.g., requiring data analysis projects), and offering more courses on advanced research methods (e.g., natural language processing, mobile sensing, and/or social network analysis). One practical recommendation akin to training in other STEM disciplines (e.g., biology, chemistry, physics) would be to add accompanying laboratories to the core psychology topic classes and provide hands on training for making inferences using these types of methods. We believe that this is an exciting opportunity to advance our field to new dimensions.

### 4.3 Toward computational thinking

We hesitate to leave interested readers with the intuition that improved quantitative skills and the application of computational modeling are simply additional “tools” that researchers should strive to acquire in service of conducting high–dimensional psychological research. Rather, through the act of engaging in *computational thinking*, psychological researchers themselves can fundamentally change the way they approach psychological problems (Anderson, 2016; Newell, 1973).

**Figure 3:**
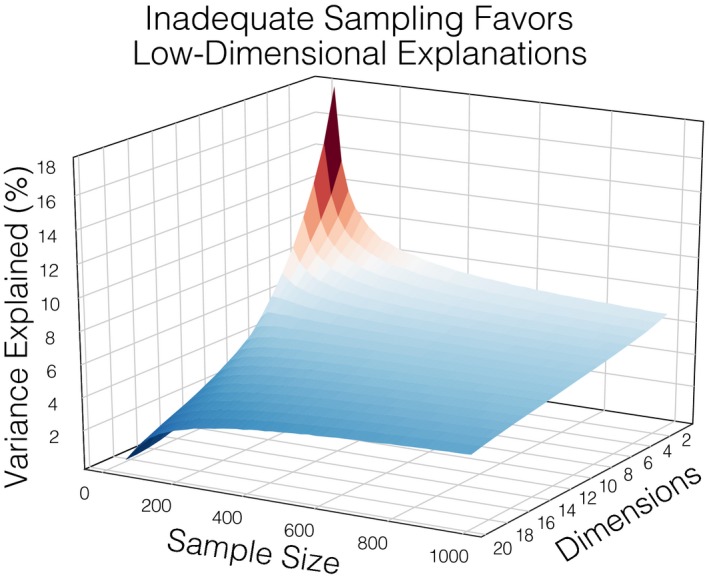
This simulation illustrates how small sample sizes are biased to favor low‐dimensional explanations even if the true underlying dimensionality of the data is high. We first generate a multivariate gaussian cloud using 10,000 observations comprised of 20 orthogonal dimensions and repeatedly draw random samples of increasing size from this space (100 repetitions per sample size). We then attempt to recover the dimensionality of the simulated data using principal components analysis and plot the distribution of variance explained across the computed components. When small samples are collected from a high–dimensional space, the majority of variance explained comes from the first few (2–3) components, which may lead researchers to mistakenly believe that the population itself is low dimensional. However, when larger data samples are collected, the amount of variance recovered across the dimensions becomes more uniform and in line with the data‐generating process, which would lead to the correct conclusion that the sampled data and therefore the population from which they came are high dimensional.

For example, in addition to the psychological explanations enumerated above, there is also a statistical explanation for the Flatland fallacy. It is well established in the field of machine learning that making predictions entails a trade‐off between minimizing bias and variance (Hastie, Tibshirani, & Friedman, 2009). Specifically, errors in predictions can be decomposed into three separate components: (a) *irreducible error* is the level of noise intrinsic to the problem, (b) *bias error* describes errors between the true data‐generating model and the learned model averaged across many data samples, characterizing the degree to which a learned model “underfits” a data sample, and (c) *variance error* reflects the learned model's sensitivity to the idiosyncrasies present within individual data samples, characterizing the degree to which a learned model “overfits” a data sample (Geman, Bienenstock, & Doursat, 1992). It has been demonstrated that in the context of small sample sizes and high‐dimensional signals, lower dimensional models associated with greater bias error can counterintuitively make *more* accurate out‐of‐sample predictions than the true high‐dimensional model of an underlying signal (Friedman, 1997). This means that there can be a computational benefit to prioritizing parsimony and bias (Gigerenzer & Brighton, 2009) when predicting complex psychological phenomenon from small datasets. It also offers an additional explanation for why so many researchers have converged on low‐dimensional accounts of psychological phenomena: Lower dimensional theories better explain small or inadequately sampled datasets. We provide a simulation illustrating this point by demonstrating that a principal components analysis of high‐dimensional data is biased to find a low dimensional solution when undersampled (Fig. 3). While psychologists might be naturally inclined to further simplify their experimental manipulations and increase their sample size to improve power, computational thinking predicts adopting an alternative strategy. Rather than reducing high bias error via collecting larger sample sizes alone, computational thinking highlights the importance of measuring psychological phenomena with greater sampling *diversity*. For example, naturalistic experiments, comprised of free‐viewing/listening to dynamic movies and unconstrained social interactions, elicit a greater range of psychological experiences (e.g., Chen et al., 2017; Haxby et al., 2011; Huth et al., 2016; Zadbood, Chen, Leong, Norman, & Hasson, 2017). By measuring and eliciting psychological phenomena in numerous ways, researchers can more richly sample high–dimensional effects of interest. It is with this data diversity that high‐dimensional models can outperform biased lower dimensional alternatives. To this end, to combat the Flatland fallacy, we believe that psychological scientists should additionally strive for large sample sizes as well as large data *diversity*, richly sampling from a larger spectrum of human experience.

## 5 Summary

In this paper, we have outlined how subjective biases, limitations of human cognition, social and cultural norms surrounding experimental design, analysis and pedagogy, and communicative shortcomings in discussing complex ideas can lead psychological researchers to consistently converge on low‐dimensional explanations of human psychology. To avoid committing this “Flatland fallacy,” we have proposed a reimagining of the field of psychology, which emphasizes a culture of developing, testing, and sharing computational models, accompanied by improved quantitative and technical training. Like cooking recipes, models provide a formal framework for taking inputs and transforming them into products (predictions). More specifically, models offer researchers a tool to assist reasoning in higher dimensional ways, approaching psychological science as an explanatory and predictive discipline (Yarkoni & Westfall, 2017), and most important, facilitating the development of a cumulative science rather than one characterized by a patchwork of disparate low‐dimensional theories (i.e., “you can't play 20 questions with nature and win” [Newell, 1973]).

We note that this is not a trivial proposal. Redesigning the entire curriculum for undergraduate and graduate training in psychology will take many years. The adoption of a computational framework will inevitably generate a host of additional complications and difficulties. For example, increasingly complex computational methods will necessarily increase the difficulty in communicating research findings. However, our methods of inquiry as scientists should not be determined by the ease with which we can communicate our work. Rather, our methods of inquiry should strive to directly tackle the immense complexity of our discipline while making it easier to collaborate, share, and develop our cumulative understanding. We believe these initial obfuscations provide a net benefit to both the general public and other scientists: They remind us that understanding how the human mind works is an incredibly challenging endeavor easily rivaling the difficulty of problems studied for decades in disciplines such as physics, astronomy, chemistry, biology, and computer science and should be accordingly approached with commensurate awe, rigor, and humility.

## Author contributions

E.J. and L.J.C. conceived of and wrote the manuscript.

## Competing financial interests

L.J.C. is supported by funding from the National Institute of Mental Health (R01MH116026 and R56MH080716).

## Notes

1 With the clever manipulation of visual properties (e.g., color, texture, animation), it may be possible to graphically represent multiple interacting factors, but as the number of factors increases, difficulties in interpretation increase dramatically.

2 This definition captures a variety of statistical and machine learning approaches, including linear and nonlinear supervised, unsupervised, and reinforcement learning models.
